# From an Intersectional Perspective Reframing Aging and Care Experiences of Taiwanese Women Caregivers

**DOI:** 10.1093/geroni/igaf122.4297

**Published:** 2025-12-31

**Authors:** Yen-Chin Lee

**Affiliations:** National Chung Cheng University, Taipei, Taipei, Taiwan (Republic of China)

## Abstract

This study applies an intersectionality framework to fundamentally reframe our understanding of aging and caregiving in Taiwanese society. By meticulously examining the complex experiences of women caring for sick parents-in-law on behalf of their husbands, it aims to expose the systemic social inequalities often obscured by homogeneous views of aging, advocating for more socially just and inclusive gerontology education and long-term care policies. Utilizing a qualitative research approach, the study conducted semi-structured, in-depth interviews with five Taiwanese female caregivers. Reflexive thematic analysis, guided by intersectionality theory, was employed to reveal how deeply ingrained traditional concepts of “male superiority” profoundly influence caregiving roles, familial power dynamics, and well-being, providing a nuanced empirical basis for reframing. The research highlights that caregivers’ experiences are shaped by intersecting power relations, key insights include: 1) the “taken-for-granted” role of daughters-in-law due to gendered labor, economic disparities and unpaid labor, 2) the multiplex burden of “double aging care” and 3) the profound impact of urban-rural and ethnic differences. Despite these pressures, caregivers demonstrated remarkable resilience and a desire for “women-empowerment” and “life-awakening”. This study calls for structural reforms in family care support, e.g., exploring “community of reciprocity and aging together”, social mutual aid mechanisms like a “collecting points to redeem a free service”, and expanding gerontology education to all age groups to foster genuine gender equality and social justice. Ultimately, it appeals for the voices of vulnerable Taiwanese women to be heard and seen to build a more equitable and supportive super-aged society.

